# Evaluating Locational Preference of Urban Activities with the Time-Dependent Accessibility Using Integrated Spatial Economic Models

**DOI:** 10.3390/ijerph19148317

**Published:** 2022-07-07

**Authors:** Asif Raza, Ming Zhong, Muhammad Safdar

**Affiliations:** 1 Intelligent Transportation Systems Research Center, Wuhan University of Technology, Wuhan 430063, China; raza@whut.edu.cn (A.R.); safdar@whut.edu.cn (M.S.); 2 National Engineering Research Center for Water Transport Safety, Wuhan 430063, China; 3 Engineering Research Center for Transportation Safety, Ministry of Education, Wuhan 430063, China

**Keywords:** land use, livable cities, urban activities, spatial economic models, sustainable development, dynamic accessibility

## Abstract

In recent years, accessibility has been considered a vital policy objective in the development of a sustainable transportation system. Accessibility can make a significant contribution to quality of life. The accessibility of desirable locations, such as households and commercial locations, is usually underpinned by land use patterns and transportation infrastructure. The accessibility of different activities is largely determined by the spatial distribution of activities and associated transport networks in a metropolitan area. In order to examine how location choice behaviors of urban activities influence urban forms and land use patterns, accessibility has been used extensively to consider the impact of both the spatial distribution of opportunities (e.g., employment or services) and the transport cost of reaching them. However, in most cases, only those static/aggregate accessibility terms that are represented by the “logsum” of all available transport modes have traditionally been used in urban transport planning and modeling analysis. In this study, according to urban activities, several Integrated Spatial Economic (ISE) models i.e., PECAS (Production, Exchange, Consumption, Allocation, System) models were developed to study the relationship between dynamic, Time-Dependent Accessibility (TDA) and the location choice behavior of urban activities in the City of Wuhan, China. The developed models were then used as tools to investigate the impact of dynamic/disaggregate short-term TDA on location choice behaviors of various urban activities such as households and commercial. Regarding the household location choice, the ISE modeling results revealed that urban households living in the downtown area of the City of Wuhan were sensitive to TDA to employment centers, especially during the morning peak time. In addition, commercial services prefer locations that offer a high level of accessibility during off-peak times. Based on the results of this study, it is recommended that planning exercises, such as the development of zoning and the allocation of urban activities and public facilities, pay more attention to dynamic, short-term TDA, which is essential for urban sectors to carry out daily activities, than their static, composite accessibility counterparts.

## 1. Introduction

Livable cities make a significant contribution to sustainable development. A livable city is one where residents enjoy a healthy lifestyle and economic prosperity. Accessibility is an integral part of livable cities, as it significantly influences the various behaviors of activities within the city, such as travel behavior and home location choice [1]. The significant impact of accessibility on sustainability and urban economics is widely acknowledged [2]. The accessibility of desirable locations (households, commercial) is usually underpinned by land use patterns and transportation infrastructure. Cities are designed to enable people’s access to other individuals, goods, and services. This is accomplished by appropriate transport networks that expedite the movement of people across space, and time and which facilitate the spatial distribution of people, goods, and services [3]. Accessibility is the ease with which desirable destinations may be reached and is thus a crucial indicator of urban efficiency [4,5]. Accessibility measures are indicators that reflect the impact of the land use patterns and transport system distribution on users. It means that both terms, land use and transportation, should be linked because they give people the opportunity to take part in activities taking place at different locations [6]. It would seem reasonable to assume that a location that is highly accessible and well served by the transport system would be more attractive to activities that prefer such accessibility (e.g., commercial activities). Highly accessible areas could be more appealing to households seeking different types of opportunities, e.g., schooling and leisure, and thus are likely to be developed into commercial or high-end residential spaces [7,8,9]. Therefore, changes to the transport system can affect the land use system, and vice versa.

In the literature on urban planning, geography, and transportation, the effect of accessibility on household locations has a long and esteemed history as a subject of study [10]. Traditional mobility-based planning tends to evaluate transport system performance primarily based on traffic speed and therefore favors automobile-oriented transport improvement [11]. On the other hand, accessibility-based planning considers additional impacts and options, including improvements to alternative modes, incentives to change travel behavior, and most importantly, more accessible land use patterns [1,12]. Many current planning practices tend to favor mobility over accessibility and automobile travel over alternative modes [13]. A comprehensive analysis of accessibility can help decision makers to identify more optimal solutions. However, evaluating accessibility is challenging. Different planning issues require different accessibility analysis methods to account for different users, modes, scales, and perspectives [9]. Accessibility to activity location (households and firms) coupled with advancements in the specification and estimation of econometric models has led to substantial progress in the area of urban planning/modeling in recent years [14]. However, the literature shows that these approaches have almost failed to incorporate subtle details of accessibility, such as those that are particularly related to a given time of the day or mode [15,16,17,18].

The fast growth of the economy and the continuous advancement of urbanization introduces many problems and challenges to cities in both developed and developing countries alike [19]. The continuous expansion of cities, the increasing demand for land, traffic congestion, and environmental pollution have begun to constrain the development of society and the economy [20]. Among them, traffic congestion has placed enormous pressure on urban development and the residential lifestyle [21]. From the traditional point of view, the leading cause of traffic congestion is the imbalance between the supply and demand of urban transport systems. The transport supply is passively adapted to the demand, which may lead to the consumption of many resources, but it still cannot meet the increasing travel demand. However, the deeper problem is that in transport planning and design, the interaction between land use, urban activity, and transport demand and supply has not been systematically considered. Most developed nations, including the United States and countries in Europe, have adopted integrated land use transport models for sustainable urban planning [22,23]. However, such models have not been developed or implemented in most megacities and emerging cities in developing nations. Therefore, in order to tackle these problems, a few cities in Mainland China have started to develop urban models that can integrate economy, land use, transport, and environmental protection plans during the planning process. PECAS is a robust modelling approach for spatial economic systems. It is designed to simulate the land use component of land use transport interactive modeling systems. It is comprised of four modules, including activity allocation, space development, transport model, and an economic and demographic aggregate forecasting model. It is built on the theories and experiences of its pioneers, MEPLAN and TRANUS [24,25,26]. The PECAS approach has been implemented in various locations for urban and regional planning around the world over the last 15 years, including California, Caracas regions, Alberta, Oregon, and San Diego [27]. Such models are used to study the relationship between transport demand and changes in economic growth, spatial distribution/location choices of socio-economic activities, and resulting land use patterns, and to predict their changes in the future [26,28]. The primary analysis tool within such models is accessibility, which is considered as the important linkage between the transport system and the land use system, and it explains how the two interact with each other over time and space [29].

The conventional location-based accessibility measures are static/aggregate in nature and cannot reflect the accessibility fluctuation resulting from the differing performance of various modes (e.g., walking, cycling, car, bus, and metro) at different times of the day [30]. This static accessibility approach has been traditionally adopted by policymakers in planning exercises due to its simplicity. In “static” accessibility studies, destination attractiveness is measured through variables such as population or employment and an average of the congested travel/transport time during the peak hours between an origin and a destination. However, accessibility studies may need to consider the changes in the destination’s attractiveness at different times of the day. The traditional accessibility terms are found to be inadequate to consider the short-term and long-term needs of urban activities. Therefore, it is important to find a solution that could provide a better planning tool to understand the urban system well.

This study aims to help untangle urban complexity by analyzing the impact of “dynamic accessibility” through Time-Dependent Accessibility (TDA) on the key component of the urban system: how time-dependent accessibility influences the location preferences of urban activity generators, such as households and firms. Dynamic accessibility considers the short-term term and long-term needs of urban activities and could be a more useful tool in urban and transport planning compared to traditional “static” accessibility terms [30]. This study focuses on dynamic short-term accessibility in order to investigate the location preferences of urban activities. This could lead to the development and use of a practical decision-making tool to analyze the impact of TDA (under different time periods and transport network impedance conditions) on the location choice behavior of urban activities, using advanced Integrated Spatial Economic (ISE) models, i.e., PECAS (Production, Exchange, Consumption, Allocation, System). Therefore, it should be reasonable to assume that such a dynamic nature of the TDA may have a much more significant impact than the traditional, static accessibility term (e.g., those using free-flow or congested travel/transport time) on the location choices of urban activities. This study will help urban planners and policymakers to better understand such an impact and therefore enhance their urban planning exercises.

The rest of the paper is structured as follows: Section 2 provides an overview of the literature regarding accessibility measures and modeling techniques. Section 3 explains the study areas, data, and methods, including the transport model and spatial economic models. Section 4 elucidates the study results. Section 5 demonstrates discussion and comparison with previous studies. Finally, Section 6 summarizes the main findings, limitations, and recommendations for forthcoming studies.

## 2. Literature Review

### 2.1. Overview of Accessibility Measures

Over the past decades, a wide range of practitioners and researchers, including transportation planners, geographical analysts, spatial economists, and policymakers, explored and employed accessibility for a variety of urban planning goals. Accessibility can be viewed from several perspectives and vantage points [31]. The literature shows that transport shapes the distribution, characteristics, and growth of different land use, since it modifies the conditions of accessibility of the various places that make up urban space. Changes to the transport system also affect the land use system and vice versa. It means that both concepts, land use, and transportation should be related, as they allow people to engage in activities taking place in different areas.

Accessibility studies have been performed in many geographical contexts around the world. From the perspective of developed nations, a study was conducted in the post-Soviet capital city of Estonia to assess the dynamic accessibility. The findings of the study suggested that the most influential aspect of accessibility was typically the activities of social practices with strict opening hours, such as the availability of stores [30]. Similarly, a recent study was undertaken in the United Kingdom to evaluate whether better public transportation job accessibility, as modeled at the disaggregate individual level, increases employment prospects for British residents. The accessibility of potential job locations via public transportation could increase the likelihood of employment, particularly in metropolitan areas, smaller cities and towns with lower car ownership rates, and low-income neighborhoods [32]. Another study was undertaken in Indiana, United States, to investigate the effects of personalized accessibility information on residential location selection and travel behavior. The findings indicated that personalized accessibility information can influence the decision-making process of re-locators regarding their new residential location. Participants in the treatment group who received personalized accessibility information were more willing to place a greater emphasis on the accessibility-related factors of potential residence locations during the residential location decision-making process, and to choose a residence in a neighborhood that was more accessible using multiple modes of transportation, as well as better suited to their particular household travel needs [33]. Likewise, Lei and Church [34] analyzed accessibility using GIS data structure to capture temporal aspects of a transit service in Santa Barbara, California. The findings indicated that travel times by public transportation varied over time; therefore, the departure time had a significant impact on the public transit network’s accessibility.

On the other hand, from the perspective of developing nations, a study was conducted in Shanghai, China to quantify public transit-based access to healthcare services via public transportation. The researchers chose census tracts in central Shanghai with less easy access to health facilities by public transit. In contrast, access to peripheral areas was sufficient, despite the absence of nearby healthcare facilities [35]. Another study was performed in Dhaka, Bangladesh to investigate the spatial accessibility of urban facilities. The results showed that Transit-Oriented Development (TOD) neighborhoods exhibited greater spatial accessibility of urban facilities compared to non-TODs. The spatial accessibility of urban facilities has also improved significantly in TOD areas over time [36]. Similarly, Almansoub et al. [37] conducted a study to evaluate the effect of accessibility on Mixed Land Use (MLU) at parcel level in the city of Wuhan, China. The study results showed a substantial relationship between the supply of public transportation and MLU. Additionally, the study results indicated that MLU was widely available in locations with high accessibility, high density, and that were close to the city center. Likewise, Bivina et al. [38] investigated the accessibility of the metro for pedestrians in Delhi, India. The findings indicated that walking access to metro stations was greatly influenced by security and safety, as well as mobility and infrastructure.

Accessibility is a term that has been around for over four decades, as stated earlier [17,39,40]. Based on the literature, the measurement of accessibility is rich. Recently, Miller [41] proposed a people-based accessibility measure in the literature. Traditionally, place-based accessibility measures are used, which include measurements of individuals’ spatial separation and specific activities. A significant improvement in transport infrastructure or facilities would lead to an increase in the accessibility of particular land use in the urban environment. A fair distribution of transport resources is essential to provide improved access to business locations such as housing, schools, and shopping centers. In order to analyze the distribution of transport services and to measure the interaction between land use and transport, opportunities for residents in a region and accessibility measures are crucial [15,42]. To understand the existing models and their components, understanding the main concepts of accessibility is essential; this requires a comprehensive review of the literature. Thus, the outcome of the review will help to identify the study gaps.

Accessibility has become of key importance in analytical measurements, and the literature is rich with accessibility applications [37,43]. It is important to understand the shortcomings and the theoretical concepts behind existing models for designing more robust accessibility models. Various measurements have been developed and introduced over the past decades. Handy and Niemeier [44] divided accessibility measures into three main types: (i) Accumulated-based; (ii) Gravity-based; (iii) Random utility theory-based. Geurs and Van Wee [45] classified four types of measures for accessibility. (i) Infrastructure-based measures: These are used to measure (observed or simulated) the efficiency or service level of the transport system (such as congestion level and average travel speed on the road network). Usually, this form of accessibility measure is used in transport planning exercises. (ii) Location-based measures: These are used to analyze the accessibility of different sites, typically at a macro-level. These measures describe the accessibility level of spatially distributed activities, for instance, access to job activities that are distributed in the space within 30 min of transport. More specific location-based strategies explicitly introduce performance limitations on the characteristics of the supplied activity to identify the impact of competition. Usually, location-based interventions are used in urban planning and spatial studies. (iii) Person-based measures: These are used to examine accessibility at the individual level, such as the activities that a person can engage in at a given time. This form of measure is established in Ilägcrstrand [46] space–time geography, which calculates the limits on the freedom of movement of an individual in the area, i.e., the place and length of compulsory tasks, the time budgets for versatile tasks, and the speed of travel that is allowed by the transport network. (iv) Utility-based measures: This type of accessibility is used to analyze the economic benefits that one can obtain by accessing the spatially distributed activities. This form of accessibility measure emerges from economic studies.

### 2.2. Modeling Techniques for Location Preferences and Interactions of Urban Activities

The first Land Use Transportation Interaction (LUTI) model was the Lowry model, which was developed by Lowry in 1964 [47]. The Lowry model combined the theory of spatial interaction with the economic basis theory of Hoyt [48], which states that there are two economic sectors within an urban system: first, the basic activities, which consider that the location is exogenous to the model; second, the non-basic activities, where the distribution depends on the location of the resident in the study area. The Lowry model was implemented in various American cities, especially in Pittsburgh, where it was originally developed. Foot [49] made one of the first published integrated model classifications, which classified the operational integrated models that were built up to the 1980s into four main types, considering the theoretical basis from which they were built. The first models are based on the spatial theory of interaction [47,50]; second, the models that are based on linear regression, such as the EMPIRIC model [51]; third, the models that are based on optimization techniques, such as the TOPAZ model [52]; and fourth, the hybrid models, which combine elements from Garin–Lowry’s model. Later, Anas [53] introduced an alternative classification to Foot. These classifications are also based on the theoretical nucleus of a model. Anas considered five types of urban and regional models for this, as follows: (i) Monocentric models: these models come from urban economics theory such as the Alonso model and its extensions. Alonso [54] is the most common example of the Monocentric model. (ii) Non-economic models: these models do not derive directly from economic theory. (iii) Models that are based on mathematical programming: optimization-based models such as the Herbert–Stevens model [55] and the Technique for Optimum Placement of Activities into Zones (TOPAZ) model. (iv) Models that are based on econometric techniques: these models are also linked to urban economic theory and estimated with statistical data using traditional econometric techniques. The NBER model [56] would be one example of an econometric model. (v) Input–output matrices-based models: these models are applied to large urban or regional areas, such as the MEPLAN [24] and PECAS [28] model.

Over the past 60 years, several LUTI models have been developed. Southworth [57] compared 17 applied models that were reported between 1985 and 1995 to show the theoretical evolution of LUTI modeling. Waddell [10] discusses the benefits of UrbanSim and compares it to the other modeling frameworks. Timmermans [58] has grouped 22 models into three categories based on the theories on which their modeling is predicated. Wegener [59] contrasts 20 models with different features, such as perception, general structure, and theoretical basis. Hunt et al. [28] compare the state of practice (represented by six LUTI modeling frameworks) with what they consider in various respects to be “ideal modeling”. Iacono et al. [60] evaluate the evolution of theoretical approaches and how complexity is represented by reviewing 18 LUTI models. Acheampong and Silva [25] present a LUTI review focusing on the challenges that are presented by the approaches to modeling and their solutions. Recently, in a generic review of LUTI model applications, Thomas et al. [61] explored the limitations of these models’ effectiveness due to their simplistic geographical approach. Most of the existing models are as follows: Integrated Model of Residential and Employment Location (IMREL) [62]; Marcial Echenique and Partners (MEPLAN) [24]; Transportation and Environment Strategy Impact Simulator (TRESIS) [63]; Microeconomic Land Use Transportation Model (METROSIM) [64]; Modelo de Uso de Suelo de Santiago (MUSSA) [65]; Production, Exchange, Consumption, Allocation, System (PECAS) [66]; Random-Utility URBAN (RURBAN) [67]; Transport and Land Use Model Integration Program (TLUMIP) [10]; Transport and Land Use Model (TRANUS) [25]; Urban Simulation (URBANSIM) [10]; and DELTA, which is an abbreviation for different five sub-models. The transition and growth model; location and property market model; employment and status and commuting model; development model; and area quality model (DELTA) [1] have detailed sub-models for metropolitan land and housing. These models use a multi-industry, multi-regional input–output system to forecast production and consumption positions in the urban area, where households of different types are viewed as labor-producing industries and commodity-consuming ones. Most of the existing operational LUTI models, particularly those that are based on spatial interaction and utility, adopt the four-step approach. The existence of at least three main categories of integrated models, based on previous LUTI modeling reviews [25,58,60], are spatial interaction models, econometric models, and disaggregate microsimulation models. The PECAS model was developed for forecasting and policy analysis at urban and regional levels in Caracas Region in Venezuela [68].

In summary, the review of the aforementioned accessibility models has revealed several drawbacks to the existing approaches. The accessibility models used a single time and distance as the essential impedance (static approach) to calculate accessibility. The location-based approaches that were used in accessibility modeling were often static. However, accessibility’s main components, such as opportunities and social activities, are dynamic and affect the location choice behavior of the activities [30]. Despite time-dependent accessibility modeling advancements, most location-based accessibility models still rely wholly or partially on a timely perception of accessibility [69,70]. These “static” models presume that household location is static and that both transport supply and opportunities for social practice activities are fixed in time and space. These models may lead to biased or even deceptive assumptions in accessibility models [71]. However, even in the case of partially dynamic location-based accessibility studies, there is often a lack of information about people’s actual whereabouts in time [72]. These models fail to capture the fine details of accessibility, especially their temporal distribution and mode distribution. The present trends in transport and urban planning are not sustainable, and changes to transport and land use systems are essential to meet these needs [73]. Therefore, this study intends to contribute to the existing literature by studying the location choice behavior of urban activities with the TDA using advanced ISE models. The advantages of this method include capturing very fine details of how the decisions of locating various urban activity generators (such as households, businesses, firms, etc.) are made. The PECAS model is a scientifically sound integrated spatial economic model which has been widely used for forecasting and policy making at urban and regional levels. However, this novel form of a spatial economic model—which represents the interactions between the transportation system and land use, and the broader spatial economic system—includes a broader range of economic impacts, such as wages, rents, productivity, consumer surplus for segments of households, and labor. This model is implemented in many regions in developed and developing countries—particularly, in California, Caracas regions, Alberta, Oregon, San Diego, Wuhan, and Shanghai. Wuhan is the first city in China to adopt PECAS for urban planning/modeling.

## 3. Study Data and Methodology

### 3.1. Study Area

**Figure 1:**
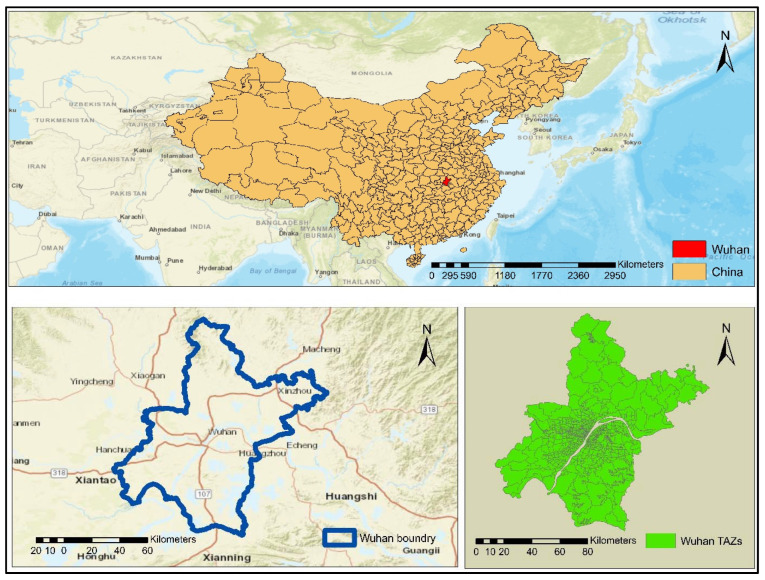
Study area, Wuhan City, China.

This study considers the City of Wuhan as a case study. Wuhan is the capital city of Hubei Province, China. The City of Wuhan had a population of 10.1 million permanent residents in 2012, including 6.83 million urban residents and 3.28 million rural residents. The population increased to 10.6 million in 2015 with an increase of 7.48 million urban residents; however, the rural population decreased to 3.11 million because of the urbanization process (http://tjj.wuhan.gov.cn/tjfw/tjnj/) (accessed on 20 March 2021). This decline may also be attributed to socioeconomic conditions, natural facilities, transportation facilities, educational facilities, and land use changes over time and space. The map of the study area (Wuhan City, China) is depicted in Figure 1.

### 3.2. Study Data

A reliable dataset is vital in the development of integrated land use transport models. The City of Wuhan is divided into 690 Traffic Analysis Zones (TAZs), and 147 Land Use Zones (LUZs). The corresponding TAZ shapefiles are obtained from the Wuhan Transportation Planning Institute (WHTPI) (http://www.whtpi.com/Default.html) (accessed on 25 June 2021). Population and employment by type and year are major datasets that are used in the development of the Transport Demand Model (TDM) and ISE models. One of the major datasets that is used in TDM and ISE models is employment data. The City of Wuhan had around 4.77 million employees working in various sectors in 2012, which increased to 5.06 million in 2015 (Wuhan statistical Yearbook) (http://tjj.wuhan.gov.cn/tjfw/tjnj/) (accessed on 28 July 2021). The employment data are obtained from the WHTPI.

#### 3.2.1. Household Travel Survey (HTS) Data

One of the major datasets for all transport infrastructure plans is the travel survey data of households. This dataset is used by all planning agencies to make transport policies and planning decisions. The HTS is a primary source of information that contains all the vital information to understand and quantify people’s traveling behavior using the actual transport network (http://www.whtpi.com/Default.html) (accessed on 15 March 2021). The total number of trips that are obtained from the HTS data is 21,171, including 11,392 walking trips, 7026 bike trips, 796 bus trips, 30 metro trips, 1039 car trips, and 888 taxi trips. The HTS is conducted in the downtown area, and only a random sample of the HTS is used for this study. Moreover, in the downtown area, most activities are within walking distance, and as a result, the proportion of walking trips is greater than that of other modes. In this study, 24 h travel records are used to develop the TDM and ISE models. This study only considers trips within the study area by major modes of transport (car, taxi, bus, and metro).

#### 3.2.2. Transport Networks

The design and development of the 2012 and 2015 TDM needs various network levels, such as road networks (2012 and 2015) and public transit networks (2012 and 2015). The road network contains link-type information, link distance, free-flow speed, link daily capacity by link type, and the number of lanes in each road link, as presented in Table 1.

The design and connectivity of public transit routes can affect travel behavior in several ways. A connected transit network provides better accessibility with possible transfer to other modes (such as bus–bus, bus–metro, metro–-bus, and metro–metro). Increased connectivity can reduce overall travel time by reducing the walking time that is required to reach transfer stations. A more extensive transit service has a positive impact on urban transport system performance (per-capita vehicle travel, congestion delays, traffic accidents, and pollution emissions. Connected transit systems encourage Transit-oriented Development (TOD). A python-based data mining tool was developed to download the bus and metro routes along with their respective stations for the years 2012 and 2015. The transit network contains line distance, service frequency, fare, walking, and transfer limits. Other relevant information is presented in Table 2.

**Figure 2:**
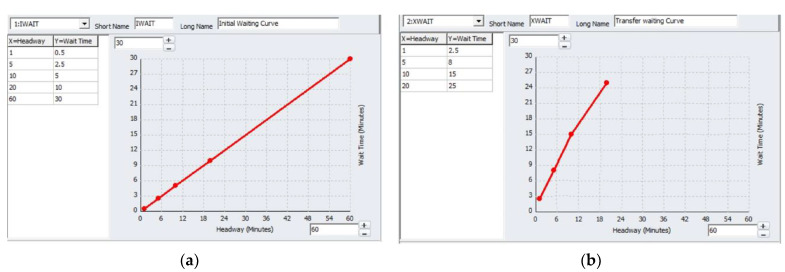
(**a**,**b**) Initial waiting and transfer waiting for curves: (**a**) initial waiting curve; (**b**) transfer waiting curve.

Table 2 presents the basic attributes of the transit system, such as the transit service frequency (or headway); static fare system for the bus and distance-based fare system for the metro (including transfer fares between different modes); and walking and transfer distance thresholds (thresholds are used to avoid the generation of unnecessary non-transit legs during route enumeration). Various curves were developed during the transit network development to calculate the waiting and transfer times, as shown in Figure 2a,b.

The initial waiting time curves were used to calculate the initial perceived waiting time (minutes) at the bus and metro stations. The initial waiting time calculated is half that of the headway, as shown in Figure 2a. Meanwhile, the transfer waiting time curve is developed to calculate the transfer waiting time at the bus and metro stations. Usually, the transfer waiting time is perceived differently compared to the initial waiting time, as shown in Figure 2b. Most commuters prefer direct routes which involve no transfer or minimal transfer waiting and walking times. The initial waiting time and transfer waiting time were used during the route evaluation process to find the best path from origin ‘i’ to destination ‘j’ with minimal walking, transfer, and travel cost.

#### 3.2.3. Land Use Data

**Figure 3:**
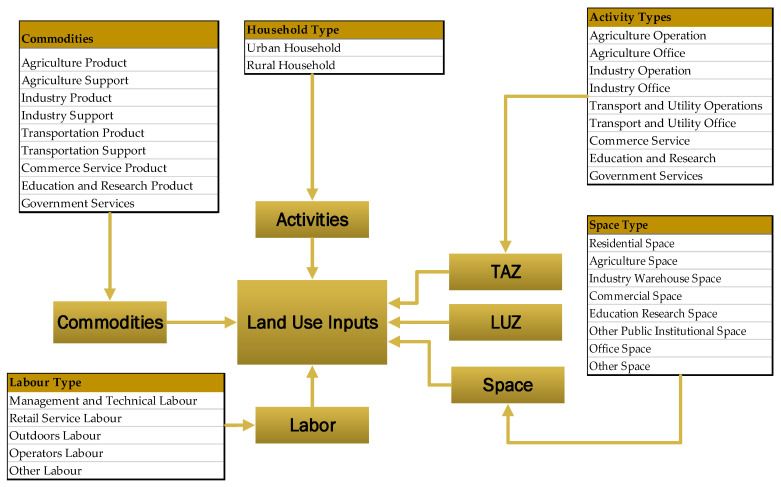
Land use input data.

The land use data in Figure 3 show aggregate spatial input–output data which represent the interaction of activities and commodity flows. These input values represent the amount of economic activity for a particular combination of sectors. This study includes various industrial activities (such as agriculture, industry, and commercial); household activities (such as urban and rural); commodity types (including agricultural products, industrial products, commercial products, and transport products); labor types (such as management and technical labor, retail labor, and outdoor labor); and space types (such as residential, commercial, and industrial). For instance, household activity produced labor and consumed various commodities during this process, and consumed residential space during the allocation process.

### 3.3. Methodology

Accessibility has a strong influence on activity locations and land use patterns. The review of accessibility models has revealed several drawbacks to the existing approaches. These models used a single time and distance as the essential impedance (static approach) to calculate accessibility. The location-based approaches that were used in accessibility modeling were often static. However, accessibility’s main components, such as opportunities and social activities, are dynamic and affect the location choice behavior of the activities [30]. Despite time-dependent accessibility modeling advancements, most location-based accessibility models still rely wholly or partially on a timely perception of accessibility [69,70]. These “static” models presume that households are at home and that both transport supply and opportunities for social practice activities are fixed in time and space, and these models may lead to biased or even deceptive assumptions in accessibility models [71]. However, even in the case of partially dynamic location-based accessibility studies, there is often a lack of information about people’s actual whereabouts in time [72]. These models failed to capture the fine details of accessibility, especially their temporal distribution and mode distribution. In this study, a multimodal transport model is developed to calculate time-dependent accessibility. Furthermore, these accessibility measures are input to integrated spatial economic models such as the PECAS model. The ISE model location/allocation is influenced by the TDA input from the TDM. The ISE models capture very fine details of how the decisions of locating various urban activities (such as households, businesses, firms, etc.) are made based on the TDA. This study used Cube Voyager version 6.5 (Bentley Systems, Incorporated, USA) and ArcGIS version 10.8 software (Esri, USA) in the developed multimodal transport network.

#### 3.3.1. Transport Model

**Figure 4:**
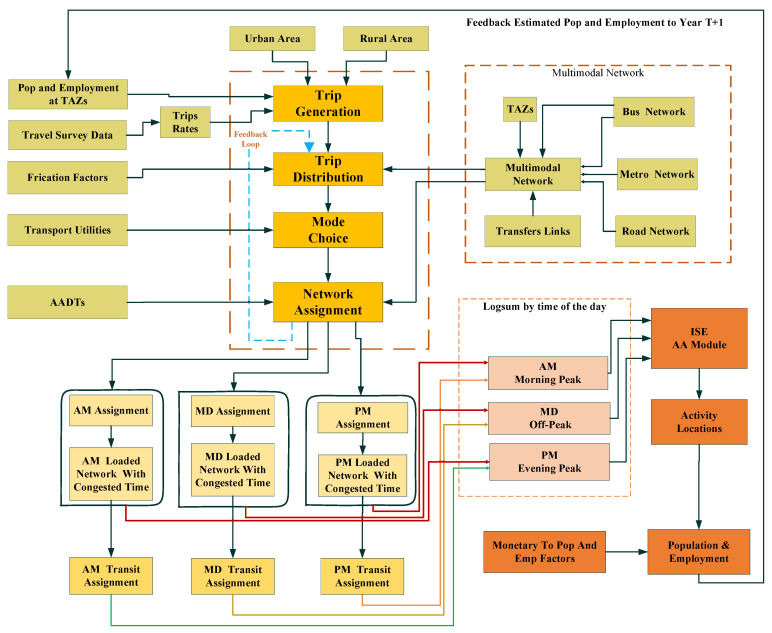
Workflow of time-dependent transport model.

Ideally, the TDM is used to calculate various time-dependent utility-based accessibility measures (time, distance, and logsum). The TDM for the years 2012 and 2015 was developed using socioeconomic data. Furthermore, the socioeconomic data were calibrated and validated for the years 2012 and 2015. The developed TDM model for the years 2012 and 2015 is used to calculate the TDA for goods, services, and other activities, as shown in Figure 4.

**Figure 5:**
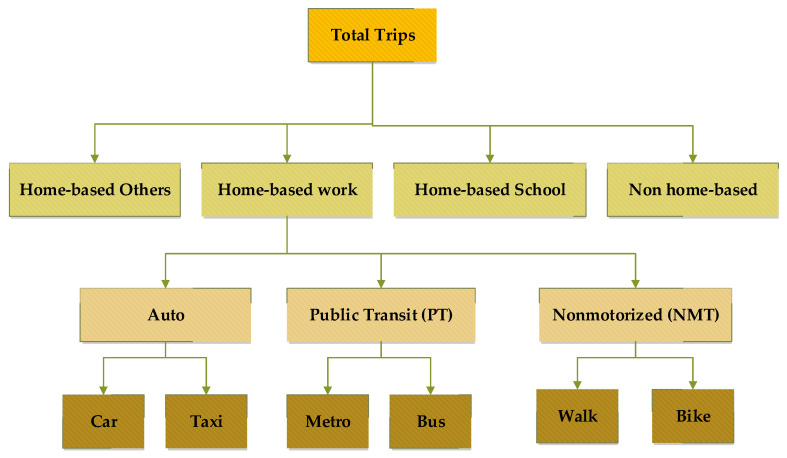
Nested logit model.

A multimodal network was developed for 2012 and 2015 using the road network, transit routes, and TAZs. The TDM development starts with the calculation of trip production and attraction rates using population, employment, and household data. The friction factor (FF) for the trip distribution was calculated using the travel survey data. Furthermore, the gamma distribution was adopted for smoothing the travel patterns before input to the trip distribution model. Transport utilities (by trip purpose and by modes) were calculated and input to the utility-based absolute nested logit model. The structure of the nested logit model is shown in Figure 5.

Furthermore, the daily model is converted to a morning peak, off-peak, and evening peak model using the hourly factors that are calculated from the travel survey data. Furthermore, the time-of-day network assignment transit model was developed to calculate time-specific congested travel time by all modes. The developed time-dependent dynamic accessibility models were used to calculate TDA. These dynamic time-dependent logsums were input to the PECAS AA module after the TDM was fully calibrated. The TDM calibration was carried out at trip generation, distribution, mode choice, and assignment level. After the TDM was fully calibrated and converged, the time-dependent utility-based accessibility measure was calculated using Equation (1).

(1) $$A_{i}=ln\overset{n}{\underset{j=1}{\sum }}e^{\left(\phi_{T}Transport_{Utij}\right)\times O_{J}\;\;\;\;\;\;\;\;\;\;\;\;\;\;\;\;\;\;\;\;\;\;\;\;\;\;\;\;\;\;\;\;\;\;\;\;\;\;}$$

where:

$A_{i}$ = utility-based accessibility measure
$Transport_{Utij}$ = transport utility (disutility) from origin *i* to destination *j* during time *t*
$\phi_{T}$ = transport coefficients (these represent the sensitivity of commuters to mode and trip type)
$O_{J}\;$= opportunities at destination zone *j* (in the case of households, jobs are considered as the opportunities, while in the case of commercial services, the residential population is considered as the opportunity).

Furthermore, the TDA (at a congested period, e.g., evening peak), which is input to the ISE model, is used to calculate the locational preferences of urban activities, as shown in Figure 5.

#### 3.3.2. Integrated Spatial Economic Model (ISE) Models

Several ISE models for morning peak, off-peak, and evening peak were developed to test the research hypothesis of this study. The components of the ISE models are:

(i) Economic and demographic module: this module consists of economic and demographic information about the study area;
(ii) Activity allocation module: the AA module of the ISE model uses nested and additive logit theory, which was adopted to test the research hypothesis of this study. The AA module is an aggregate representation of activities, commodity flows, markets with aggregate demands and supplies, and exchange prices. It concerns the quantities of activities, commodity flows, aggregate demands, and supply and exchange prices;
(iii) Space development module: the space module represents real-estate developer behavior. This module develops the space based on the exchange price signal from the AA module;
(iv) Transport module: the transport model was developed to calculate TDA to various activity locations for 2012 and 2015.

**Figure 6:**
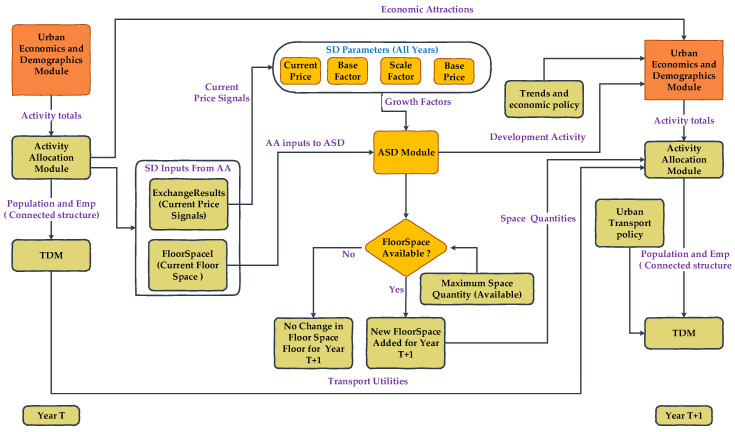
ISE model design diagram.

The ISE models based on TDA are developed based on the workflow that is presented in Figure 6. The ISE models based on TDA start with the development of the economic and demographic module. The activity location module was developed using model-wide activities and employment by industry type data. The transport module of the ISE model was developed exogenously. The Aggregate Space Development (ASD) module was developed to generate floor space (e.g., residential and commercial) for different activities (e.g., household and commercial). The ASD space module represents real-estate developer behavior. This module develops the space based on the exchange price signal (Exchange Results) from the AA module. The formulation of ASD is given in Equation (2).

(2) $$F=max\left[\;1,\;\;\;\;\;\;F_{b}+\exp\left(\frac{\left(P*\beta -P_{b}\right)}{P_{b}}\right)\right]$$

where the (*F*) Factors are applied to the current space. The model estimates the “*F*” factors for the year T + 1 based on the model input parameters that are acquired from the observed data. For instance, when the estimated factor for residential floor space is 1.06 and the current floor space is 1200 sqrt meters, then the ASD model generates 1200 × 1.06 = 1271 sqrt meters for the year T + 1. The base factor (*F_b_*) represents the starting base for the factors, while scale factors (β) represent the smoothness of the percentage increase. Both values should be greater than 0 and less than 1. The Activity Allocation (AA) Module of ISE generates the current prices for different floor space types in each TAZ. These prices from AA act as the current price (*P*) in the ASD module. Based on the current price, the ASD module generates new floorspace for the Year T+1. The base price (*P_b_*) is the average observed price. This may vary depending on the type of floor space. The ASD model checks the maximum space that is available (Maximum Space Quantity) in a TAZ before developing space in that TAZ, in order to avoid overestimation. If the specific space type is not available in the current TAZ, the ASD moves to the next TAZ where space is available for development, and then develop that space based on Equation (2).

Activities produce commodities and then transport and sell those commodities; after buying and transporting them, they consume commodities. There are various types of activities, including the industrial, government, and household sectors. The quantities of activity in the model can be measured in values (Chinese Renminbi—RMB) for business and other activities, or numbers (household). As part of its allocation process, the AA module allocates the study-area wide quantity (activity totals) of each activity among the TAZs. Accessibility plays a crucial role in land use transport interaction, linking the transport system to economic activities. A transport system’s primary function is to provide access to other people and companies so that they can participate actively in all kinds of spatially and temporally distributed activities (social, economic, etc.) and exchange information, goods, and services in a physical manner [74]. The activity allocation module represents activity locations that occur as a result of the location choice behavior of activities.

The considered locations were TAZs that were large enough to be distinct markets for activities and space. The relation between TDA and the location choice of activities is well understood using ISE models. To evaluate the locational preferences of activities using TDA, it is important to understand which time of day influences the locational preferences of activities more. The time of day contains a combination of the following hours:

- Morning peak: 7 a.m.–9 a.m.;
- Off-peak: 10 a.m.–1 p.m.;
- Evening peak: 4 p.m.–7 p.m.

Table 3 presents the time of day that is considered for both the production and consumption of each activity.

#### 3.3.3. Simulating Activity Location Choices with the ISE Model

The complex urban systems are composites of urban activities, the land/space for hosting them, and the interactions or exchanges among them. The exchanges among the urban activities are featured with the commodity (including goods, services, labor, and land/space) flow from where they are produced to where they are exchanged (from the seller to the buyer), and then from where they are exchanged to where they are consumed, with the land and space consumed at the same place that they are produced.

The movement of these flows of commodities from where they are produced to where they are consumed is the economic basis for travel and transport in the modeling system. It is the travel conditions, distances, costs, times, and associated (dis)utilities for the movement of these commodities that result in the influence of the transport system on the interactions among activities and the attractiveness of locations for activities. The AA module allocates the flows of commodities from the production location (represented by TAZ in this study) to the exchange location, and from the exchange location to the consumption location, and finds the corresponding set of prices at the exchange locations that clears all markets, as part of its allocation process.

The random utility approach was used to simulate the location decisions of the activities (household and commercial). It is postulated that the agents (households, businesses, and firms) assign a utility to each zone and choose the one that maximizes it. In the ISE system, the utility is composed of the systematic part (household and commercial activities) and a random part. Then, the utility of this joint choice is calculated using Equation (3).

(3) $$Utility_{d,k}=\frac{1}{\lambda }ln\overset{n}{\underset{z=0}{\sum }}e^{\lambda \left(\phi_{T,d}Transport_{z,k,d,T}+\phi_{p,d}Price_{z,d}+\frac{1}{\lambda }lnSize_{z,d}\right)}$$

— *d* = buying (consuming) or selling (producing) the commodity
— *k* = index for the zone of production or consumption of the commodity
— *z* = index for an exchange zone
— λ = dispersion parameter for the exchange location choice for the commodity
— $Utility_{d,k}$ = accessibility for a commodity for direction *d* and zone *k*
— $Size_{z,d}\;$= an indicator of the relative amount of the commodity offered in exchange zone *z*
— $\phi_{T,d}\;$= transport cost coefficient
— $Transport_{z,k,d,T}\;$= transport cost between *z* and *k* for *d* = buying and selling, *T* = time of the day
— $\phi_{p,d}\;$= price coefficient (always set to 1 for *d* = selling and −1 for *d* = buying because the utility is in monetary units)
— *Price z* = price of a commodity in *z*
— *Ln* = natural log

The $Utility_{d,k}$ contains three components:

(i) Transport component (cost of transporting commodities to or from the exchange zone);
(ii) Price (prices of commodities in the exchange zone);
(iii) Size (relative size of the exchange zone).

When the commodity is labor, the term wages is used instead of prices. According to the theories that are used by the ISE model framework, it is these three components that drive the decision making in the spatial economic system. These components are the reason why households and industries behave in a certain way in specific geographical and temporal contexts, and why the root of the policy investigation is centered on how the changes in these utility components are responsible for the changes in consumer surplus for a given amount of consumption of a particular commodity. These changes impact the exchange location choice, production/consumption technology choice, and home location choice of both the consumers and producers of commodities, whether they are households or industries. The utility of transporting a unit of each commodity is based on zone-to-zone measures of transport attributes from the TDM.

Activity categories are sensitive to changes in commodity utilities, and each utility component (time of the day, price, and size) could influence the other components. For instance, changes in transport costs could affect accessibility in different locations, which would eventually cause price changes for all types of commodities (including goods, services, labor, and space/land) and activity sizes across locations.

## 4. Results

### 4.1. Numerical Analysis

The developed ISE models were used to determine the relationship between TDA and the location preferences of urban activities. Usually, households are producers of labor, and these laborers are consumed by services and industries. Likewise, services and industries are the producers of jobs, and these jobs are consumed by households. The location choice behavior of activities was further classified:

— Locational preferences of household activities;
— Locational preferences of commercial services (household obtained services such as retail).

In principle, most economic activities are dependent on an acceptable level of accessibility in order to survive and develop, so a range of accessibility measures need to be considered. As mentioned earlier, a TDM was developed, validated, and calibrated for the years 2012 and 2015 to calculate TDA to various activity locations. This TDA measure is used to calculate the short-term dynamic access to goods, services, and other activities that are located in the City of Wuhan during the morning peak, off-peak, and evening peak. However, the TDA measure is limited to certain times of the day. The TDA considers only the major modes of transport (car, taxi, bus, and metro), excluding walk and bike modes, which do not influence traffic congestion. Commodity flow from production location to consumption location influences the transport system. The movement of these activities makes the location more or less attractive for households, firms, and businesses. The transport cost coefficients for the commercial services commodities and the labor commodities for the ISE model were based on local data. These coefficients may vary depending on the commodity type that is transported, for instance, it could be different for households and goods. These coefficients are calculated by dividing the vehicle operating cost per unit of the commodity per unit of distance by the money value of the commodity load on the truck.

As mentioned in the methodology, an integrated land use transport system used a composite accessibility approach to calculate the access to goods, services, and other activities that were located in the study area, which needed transport cost coefficients to determine the cost of transporting each commodity type. Furthermore, the composite accessibility measure consists of three major components that are associated with the utility of buying or selling a given commodity in a given zone.

**Figure 7:**
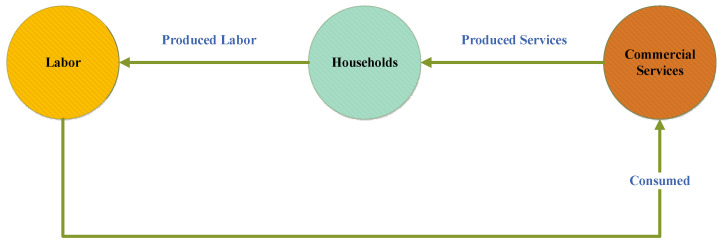
Location choice behavior of activities.

As discussed earlier, the three components of utility play a vital role in driving the decisions in the ISE spatial economic system. These three components influence the location choice behavior of households, firms, and industries. The change in the utility function influences the consumers and producers of the given commodity for households and other activities because these activities are susceptible to commodity utilities. These transportable commodities include goods, services, and labor. The categorization of goods and services is made by grouping the goods and services that are associated with the predefined industries. These all interact with each other through a transport system. Households are social units; usually, they provide labor and consume goods and services, along with space. Meanwhile, industries, firms, and businesses offer and consume goods and services, and they are the consumers of labor and space, as shown in Figure 7. Usually, households produce labor and these labors are consumed by commercial services. During the process, commercial services consume labor and produce services, and these services are consumed by households in the region.

Table 4 shows the activities and commodities and their production and consumption process. The connectivity between TAZs is based on the congested network of the transport model (time, distance, logsum, etc.), which in transport terms is called disutilities. Disutilities are used to establish the interaction between TAZs for the buying and selling of various commodities. The trips in the system are linked to the economic flow system using the calculated transport coefficients, which reflect the money cost per trip for goods, services, and labor. Due to the lack of truck trip data, the auto is used as a proxy for trucks for transporting goods. Undoubtedly, residents prefer locations with good access to utilities, different services, primary public transport systems, and employment centers.

The primary hypothesis is that residents prefer a location that is close to their place of work, or a place with a high level of access to their primary needs. Resultsrevealed that the actual influence of residential location and accessibility of the desired job location during peak and off-peak periods may depend mainly on the underlying geographical location (spatial) and temporal period (time of the day), as well as the average price of the location.

Overall, the R^2^ for the models shows a strong relationship between TDA and residential locations where the adjusted R^2^ value was found to be higher than 0.90. The results revealed that there was a significant improvement in the residual square from the years 2012 to 2015, due to the improved level of accessibility in the year 2015, as shown in Table 5. Furthermore, results showed that household locations were sensitive to morning peak TDA where the R^2^ value was found to be higher than 0.90.

Table 6 presents results for the TDA to commercial activities. Overall, the results showed that there was a strong relationship between TDA and commercial locations, where the adjusted R^2^ value was found to be greater than 0.91 for the year 2012 and greater than 0.93 for the year 2015. Moreover, the results indicated that TDA to commercial locations had a high R^2^ value of 0.98 during off-peak time. Meanwhile, there was a significant improvement in the residual square from the year 2012 to 2015 because of improved TDA during 2015.

Accessibility indexes (logsum) were used to present the TDA levels at each TAZ. For instance, “No” refers to no accessibility; “Low” refers to low accessibility that ranges between 0.22 and 0.92; “low-medium” refers to accessibility levels that are higher than low accessibility and lower than medium accessibility and ranges between 0.93~1.13; “medium” refers to a medium level of accessibility that ranges between 1.14 and 1.28; “medium-high” refers to accessibility that is higher than medium level and lower than high level and ranges between 1.29 and 1.39; and finally, the high accessibility index shows high accessibility in each TAZ and ranges between 1.40 and 1.51 during the years 2012 and 2015.

Meanwhile, the ISE model-estimated location (converted to density) is used to represent the activity locations. For instance, “No” refers to no activity locations; “Low” refers to a low level of activity locations and ranges between 0.003 and 0.004; “low-medium” refers to activity locations that are higher than lower level and lower than medium level and ranges between 0.005 and 0.008; “medium” refers to a medium level of activity locations and ranges between 0.009 and 0.013; “medium-high” refers to an activity location level that is higher than medium level and lower than high level and ranges between 0.014 and 0.021; and finally, the high activity locations index shows high activity locations in each TAZ and ranges between 0.022 and 0.038 during the years 2012 and 2015. These indexes are used to represent the TDA and activity location in each TAZ, as shown in Table 7. The TDA to household and commercial activities are calculated using the logsum indexes [75]. The TDA Logsum values are computed using Equation (1), and the activity location density is computed using Equation (3).

#### 4.1.1. Locational Preferences of Household Activities

**Figure 8:**
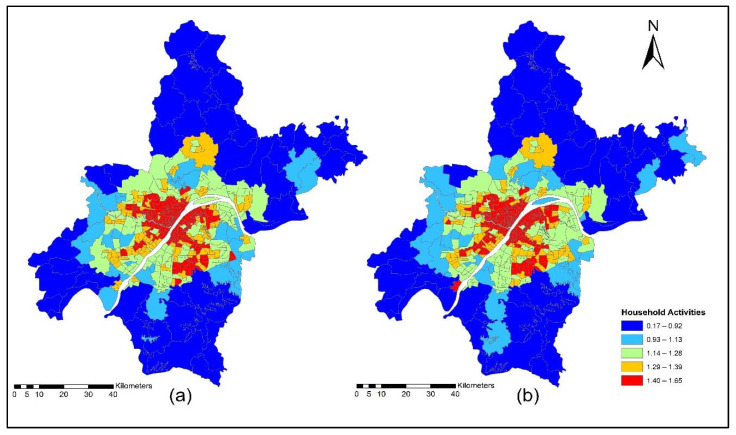
(**a**,**b**) Accessibility of household activities: (**a**) during morning peak for the year 2012; (**b**) during morning peak for the year 2015.

Households produce labor and are consumed by businesses, firms, and other industries. Except for households, all production, consumption, import, export, and labor wages are defined in terms of RMB (monetary unit). The commuting costs are calculated during the model run, and labor wages are adjusted to match the supply and demand in each location for each occupation. The categorization of employment by industry was made based on occupation in each industry. This study used multi modes (car, taxi, metro, and bus) to calculate TDA for household and commercial activities. Figure 8a,b indicate the accessibility of household activities during the morning peak for the years 2012 and 2015. The morning peak accessibility of household activities value ranges are low accessibility (0.22~0.92); low-medium accessibility (0.93~1.13); medium accessibility (1.14~1.28); medium-high accessibility (1.29~1.39); and high accessibility (1.40~1.65). The reason for high accessibility in downtown areas is the availability of multi-modes for commuting during morning peak times. In general, when the transport cost increases, the size component (accessible area) for some zones decreases for the consumer of the goods and services. All the exchanges (zones and commodities) become less accessible for the consumers, meaning that the buyers obtain less variety in the product or service. For labor, it is expected that an increase in transport can decrease the average trip length of commuting to work. Benefits losses arise due to the increase in transport disutility, but this also affects the size component, since households lose variety in job options, decreasing their size component (due to the loss in variation).

**Figure 9:**
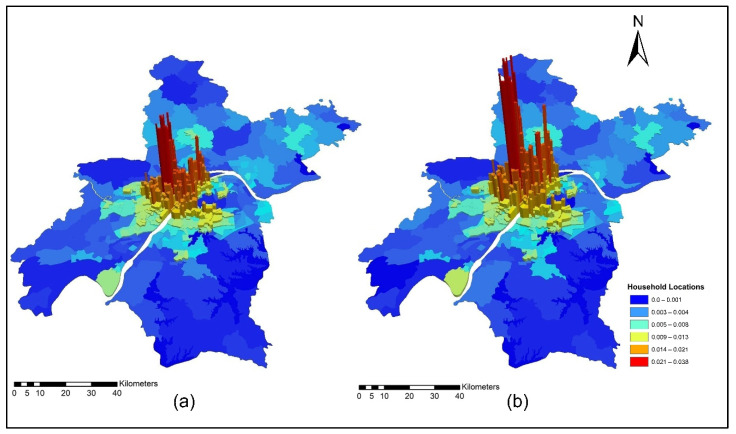
(**a**,**b**) ISE-estimated household locations: (**a**) during the morning peak for the year 2012; (**b**) during the morning peak for the year 2015.

Figure 9a,b present ISE-estimated household locations using morning peak accessibility for the years 2012 and 2015. The ISE-estimated household location density values using morning peak accessibility ranges are low household locations (0.003~0.004); low-medium household locations (0.005~0.008); medium household locations (0.009 ~0.013); medium-high household locations (0.014~0.021); and high household locations (0.021~0.038). The results also revealed that the downtown area had a high level of TDA during the morning peak. This is mainly attributed to the fact that the frequency of transit during the morning is high to help employees and workers arrive on time to their workplaces.

**Figure 10:**
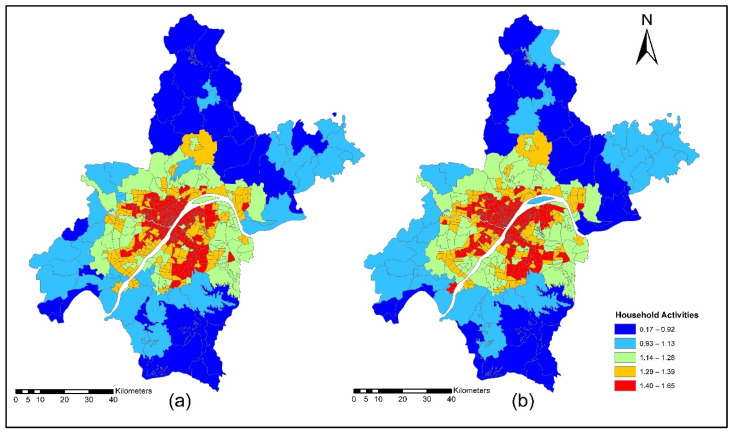
(**a**,**b**) Accessibility of household activities: (**a**) during off-peak for the year 2012; (**b**) during off-peak for the year 2015.

Figure 10a,b present the accessibility of household activities during the off-peak period for the years 2012 and 2015. The off-peak accessibility of household activities value ranges are low accessibility (0.22~0.92); low-medium accessibility (0.93~1.13); medium accessibility (1.14~1.28); medium-high accessibility (1.29~1.39); and high accessibility (1.40~1.65). Most of the retail services activities are located in the downtown area; during off-peak, the downtown area shows high TDA. The off-peak presents the low level of congestion on transport infrastructure and all activities which are sensitive to time and finds these to be very attractive.

**Figure 11:**
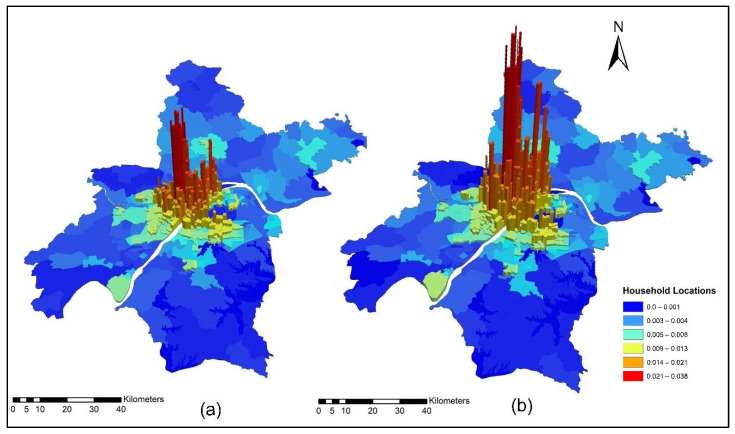
(**a**,**b**) ISE-estimated household locations: (**a**) during off-peak for the year 2012; (**b**) during off-peak for the year 2015.

Figure 11a,b present ISE-estimated household locations using off-peak accessibility for the years 2012 and 2015. The ISE-estimated household locations density value ranges using off-peak accessibility are low household locations (0.003~0.004); low-medium household locations (0.005~0.008); medium household locations (0.009~0.013); medium-high household locations (0.014~0.021); high household locations (0.021~0.038); and values that are below 0.001 represent no household locations.

**Figure 12:**
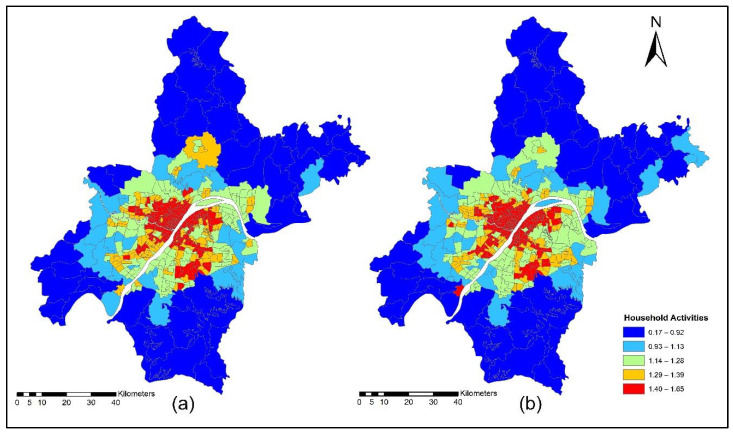
(**a**,**b**) Accessibility of household activities: (**a**) during evening peak for the year 2012; (**b**) during evening peak for the year 2015.

Figure 12a,b present the accessibility of household activities during evening peak for the years 2012 and 2015. The evening-peak accessibility of household activities value ranges are low accessibility (0.22~0.92); low-medium accessibility (0.93~1.13); medium accessibility (1.14~1.28); medium-high accessibility (1.29~1.39); and high accessibility (1.40~1.65). During the evening peak, the level of accessibility of household activities is low compared to the morning peak and off-peak time, as shown in Figure 12a,b.

**Figure 13:**
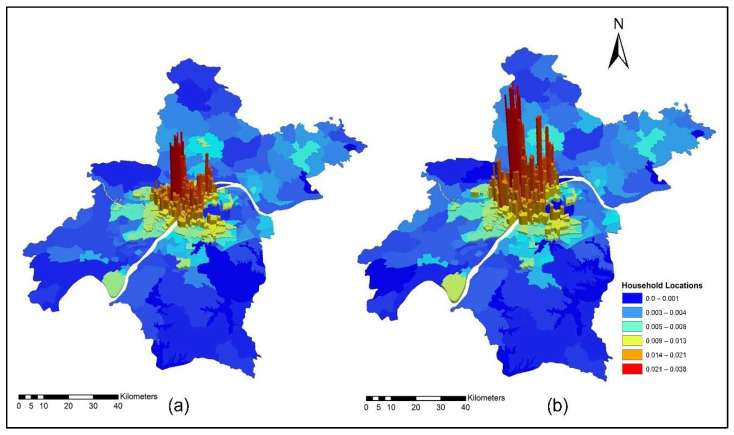
(**a**,**b**) ISE-estimated household locations: (**a**) during evening peak for the year 2012; (**b**) during evening peak for the year 2015.

Figure 13a,b present ISE-estimated household locations using evening peak accessibility for the years 2012 and 2015. The ISE-estimated household locations density value ranges using evening peak accessibility are low household locations (0.003~0.004); low-medium household locations (0.005~0.008); medium household locations (0.009~0.013); medium-high household locations (0.014~0.021); high household locations (0.021~0.038); and values that are below 0.001 represent no household locations. The household activity locations also dropped significantly because of low TDA for these activities during highly congested periods, as depicted in Figure 13a,b.

#### 4.1.2. Locational Preferences of Commercial Activities

Commercial services or business service locations are essential in any economic system. Usually, households produce labor and consume goods, services, information, retail hospitality, real estate, business services, technical services, environmental services, and private services. However, an aggregate ‘commercial activity’ term is used to present commercial services activity in the ISE system.

**Figure 14:**
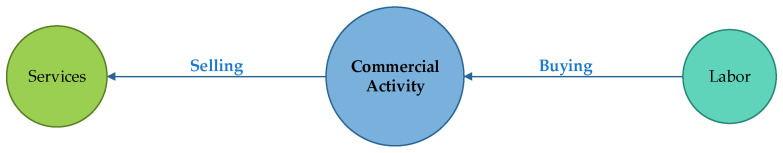
Buying and selling process of commercial activities.

Figure 14 presents the buying and selling process of commercial activities. Commercial activities usually buy labor and sell services to other activities including households. During this process, commercial activities consume various goods and services.

**Figure 15:**
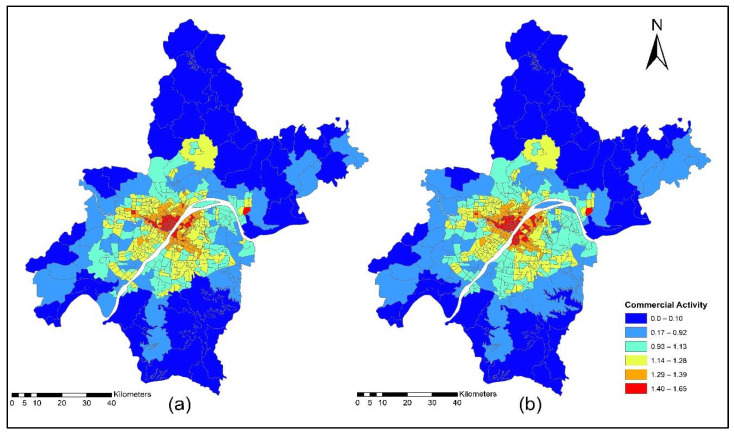
(**a**,**b**) Accessibility of commercial activities: (**a**) during morning peak for the year 2012; (**b**) during morning peak for the year 2015.

**Figure 16:**
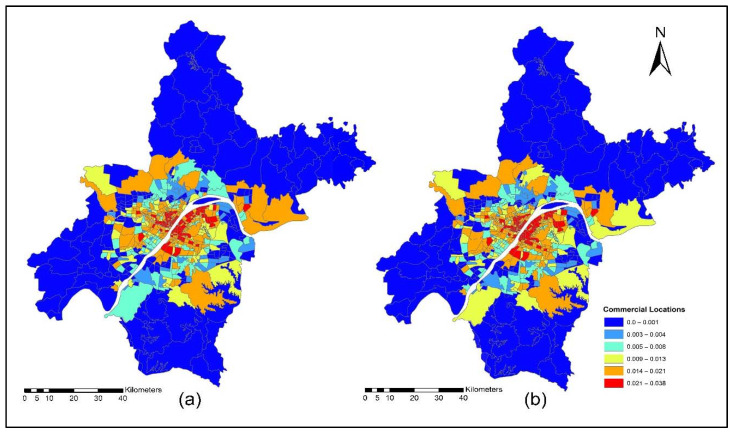
(**a**,**b**) ISE-estimated commercial locations: (**a**) during the morning peak for the year 2012; (**b**) during the morning peak for the year 2015.

The improvement in the road network and transit services improved the level of accessibility and the location choice behavior of commercial services, with better accessibility attracting more commercial activities. Commercial activities (household obtained services) prefer locations with low transport costs, which means that they only need to pay low transport costs. However, some services are sensitive to morning peak time accessibility, and some services are sensitive to off-peak time accessibility, depending on the type of commercial activity. Figure 15a,b present the accessibility of commercial service activities during the morning peak for the years 2012 and 2015. The morning peak accessibility of commercial service activities value ranges are low accessibility (0.22~0.92); low-medium accessibility (0.93~1.13); medium accessibility (1.14~1.28); medium-high accessibility (1.29~1.39); and high accessibility (1.40~1.65). The results in Figure 15a,b present that during the morning peak time, the level of accessibility of commercial activities, especially in the year 2015, is relatively high compared to the 2012 morning peak. Figure 16a,b present the ISE-estimated commercial locations using morning peak accessibility for the years 2012 and 2015. The ISE-estimated commercial locations density value ranges using morning peak accessibility are low commercial locations (0.003~0.004); low-medium commercial locations (0.005~0.008); medium commercial locations (0.009~0.013); medium-high commercial locations (0.014~0.021); high commercial locations (0.021~0.038); and values that are below 0.001 represent no commercial locations. Meanwhile, the results in Figure 16a,b also revealed that areas with high TDA to commercial services showed a high density of commercial locations.

**Figure 17:**
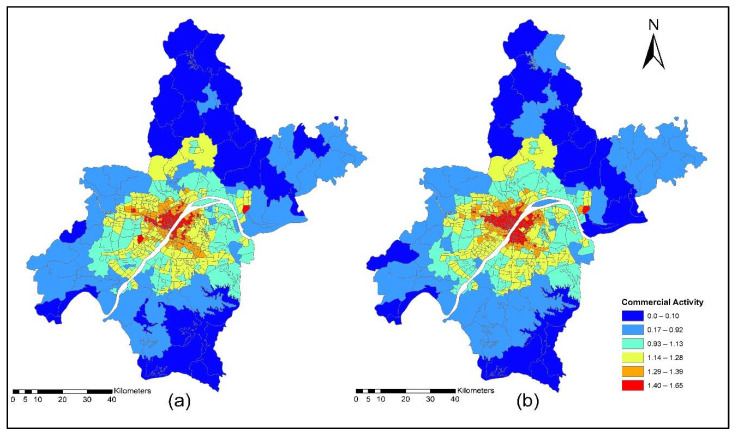
(**a**,**b**) Accessibility of commercial activities: (**a**) during off-peak for the year 2012; (**b**) during off-peak for the year 2015.

Figure 17a,b present the accessibility of commercial service activities during the off-peak period for the years 2012 and 2015. The off-peak accessibility of commercial service activities value ranges are low accessibility (0.22~0.92); low-medium accessibility (0.93~1.13); medium accessibility (1.14~1.28); medium-high accessibility (1.29~1.39); and high accessibility (1.40~1.65). Figure 17a,b present the TDA to commercial activities during the off-peak period. Results revealed that during 2015 the level of accessibility of commercial activities located in downtown and central business areas of rural zones had a high TDA.

**Figure 18:**
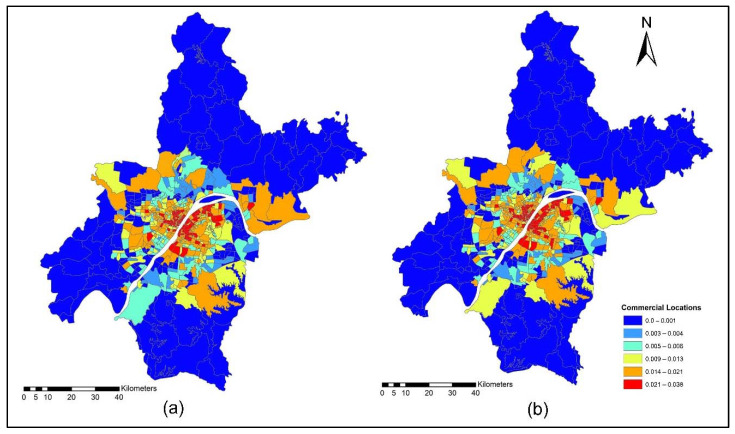
(**a**,**b**) ISE estimated commercial locations: (**a**) during off-peak for the year 2012; (**b**) during off-peak for the year 2015.

Figure 18a,b present the ISE estimated commercial locations using off-peak accessibility for the years 2012 and 2015. The ISE estimated commercial locations density value ranges using off-peak accessibility: low commercial locations (0.003~0.004), low-medium commercial locations (0.005~0.008), medium commercial locations (0.009~0.013), medium-high commercial locations (0.014~0.021), high commercial locations (0.021~0.038) and values below 0.001 represent no commercial locations. The ISE off-peak time activity location model revealed that commercial activities are susceptible to TDA as shown in Figure 18a,b. However, some commercial activities prefer and are sensitive to morning peak time accessibility, and most of them are sensitive to off-peak time accessibility. The results revealed that during the off-peak time, the level of accessibility of these services is relatively high compared to the morning peak.

**Figure 19:**
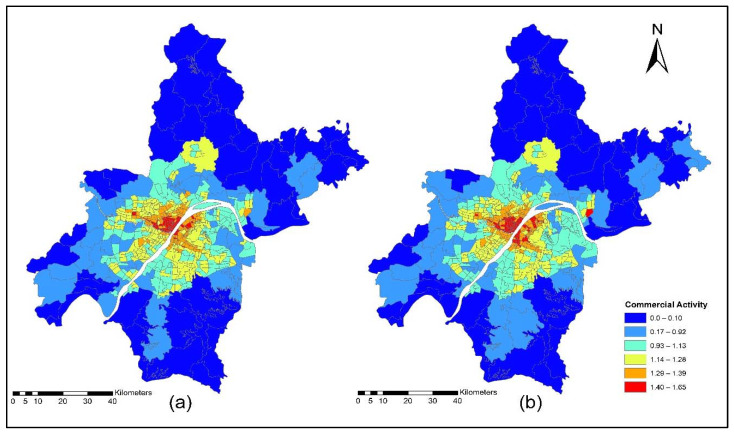
(**a**,**b**) Accessibility of commercial activities: (**a**) during evening peak for year 2012; (**b**) during evening peak for year 2015.

Figure 19a,b present the accessibility of commercial service activities during the evening peak period for the years 2012 and 2015. The evening-peak accessibility of commercial service activities value ranges are low accessibility (0.22~0.92); low-medium accessibility (0.93~1.13); medium accessibility (1.14~1.28); medium-high accessibility (1.29~1.39); and high accessibility (1.40~1.65). The commercial TDA and ISE model results revealed that during the evening peak, the accessibility of most of the household obtained services was low compared to the morning peak and off-peak time, as shown in Figure 19a,b.

**Figure 20:**
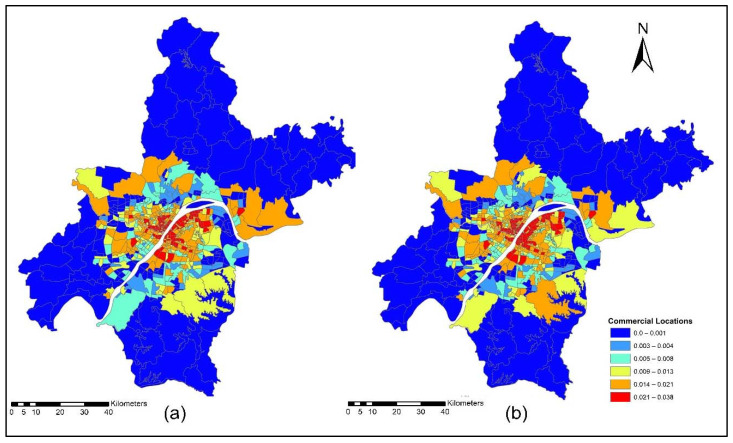
(**a**,**b**) ISE-estimated commercial locations: (**a**) during evening peak for the year 2012; (**b**) during evening peak for the year 2015.

Figure 20a,b present ISE-estimated commercial locations using the evening peak accessibility for the years 2012 and 2015. The ISE-estimated commercial locations density value ranges using evening peak accessibility are low commercial locations (0.003~0.004); low-medium commercial locations (0.005~0.008); medium commercial locations (0.009~0.013); medium-high commercial locations (0.014~0.021); high commercial locations (0.021~0.038); and values that are below 0.001 represent no commercial locations, while values that are below 0.001 represent no household locations. However, during 2015, the level of accessibility of household obtained services increased compared to 2012. This is because, during 2015, new highways were built, new bus routes were added, and a new subway line was put into service which eventually increased the level of accessibility. With increased TDA, in the year 2015, more commercial services moved to high accessibility locations, as shown in Figure 20a,b. The ISE commercial services model activity locations results revealed that commercial services were susceptible to TDA.

## 5. Discussion

Accessibility plays a vital role in social and environmental factors. The impact of location rents is poorly known. Activities tend to be close to highly accessible locations (which in turn influences rents as well). However, it is crucial to understand the actual effect of TDA on the location choice behavior of activities and decisions about where to live, where to shop, and where to work.

The time-dependent utility-based accessibility index provides a synthetic measurement of the ability to reach a particular type of opportunity within a specific time from a place of origin. Thus, accessibility is defined as a measurement of the capacity to communicate between human activities or settlements using a determined transport system. The usual measurement units are distance, time, mode, and the number of opportunities (activities). Measures of accessibility are indicators reflecting the impact that land use distribution and the characteristics of the transport system have on the users. It means that the terms, land use, and transport ought to be related, because they give people the opportunity to participate in activities that occur in different places.

The location utility of an activity that consumes a large amount of a commodity is influenced by the utility of buying that commodity. To understand and distinguish the location choice of various activities, the following models are developed using ISE models: —

— Household activity location model;
— Commercial services activity location model.

The location choice behavior of household activities depends on the nature of employment type and access to specific employment locations by a specific time of the day using specific modes. The location preference of household activities may vary depending on the household decision. Working households with no other priorities preferred a location that had excellent transport access at a specific time of day, in order to reach their job location.

The ISE results revealed that urban households living in the downtown area of the City of Wuhan were sensitive to TDA that was offered during the morning peak. It also revealed that urban households in the year 2015 showed high household activities during the morning peak. This means that urban households living in the downtown area are susceptible to TDA that is offered during morning peak time. This result is consistent with a previous study that used an expanded geographical weighted regression model, geographically and temporarily weighted regression (GTWR), and found that activities prefer highly accessible locations—such as downtown area—to employment locations during the morning peak, off-peak, and evening peak periods [76]. In contrast, several studies demonstrated that commuting occurs frequently during off-peak hours. Constant travel time metrics may overestimate accessibility, undermining the credibility of accessibility studies. This overestimation may lead commuters to underestimate their travel time; consequently, they may not reach their destinations on time [77,78]. The result of the current study also contradicted a previous study that examined urban accessibility in Madrid, Spain. The results indicated that during the morning peak time, suburban residential zones had the largest decrease in accessibility, since most inbound transport generated severe congestion problems and the population tended to be concentrated in the downtown area [79].

The results indicated that commercial activities were sensitive to TDA during the morning peak period for the years 2012 and 2015. These commercial activities, such as healthcare, social welfare, retail services, business, finance, administration, and community service, are sensitive to morning peak hours because they provide services to households and must deliver on time, particularly during the morning peak. Meanwhile, in the year 2015, when the new transit routes were added and improved the transit accessibility of commercial activities, it was found that commercial activities relocated to a location that offered high transit accessibility during the morning peak. The current study’s findings are consistent with a previous study that analyzed the spatial-temporal pattern of retail potential accessibility in Hakata station, Japan. The findings showed that the peak hour was the prime of commercial activity due to the number of transport passengers and the hours of operation of all retail services. Even though the peak hour is a prime time for retailers, its competition value is lower than that of the morning peak hour because workday finish times are more varied and relaxed than the morning time [80]. Li et al. [81] evaluated the time-varying accessibility of senior centers by public transit in Philadelphia, United States, and revealed that accessibility reduced from the city center to the suburbs. It was also found that areas with a higher proportion of elderly people tended to have less convenient access to senior centers. The results also indicated that the location decisions of commercial activities were influenced by TDA that was offered during the morning and off-peak. The result of the current study is in line with a previous study that examined urban accessibility in Madrid, Spain. The findings revealed that during the afternoon time, the transport zones in the center and north, which contained the highest proportion of jobs, were highly influenced by traffic congestion because most of the outbound journeys occur at this time [79].

Nevertheless, the results indicate that commercial and household activities prefer locations with high accessibility during different times of the day. Of course, in many cases, commercial activities are willing to pay high rent (compared to households) for location (competing for location). However, zoning rules and regulations do not allow certain activities in certain zones, or they limit their scale.

The static and ISE-TDA models results revealed that household and commercial activities preferred locations that offered high TDA, rather than static accessibility, as shown in Table 8, where most of the R^2^ values for ISE-TDA models were higher than 0.97, and most of the R^2^ values for static models were lower than 0.72. The ISE models result indicates that the location choice behavior of activities has a strong relationship with TDA and a weak or causal relationship with static accessibility.

## 6. Conclusions and Recommendations

### 6.1. Conclusions

This study aims to evaluate the impact of time-dependent accessibility (TDA) on the locational preference of household and commercial activities in the City of Wuhan, China. The spatial distribution of activities across a city results from various urbanization processes, such as increasing population/employment, change in technologies (especially those of transport), land/space development, and the development of transportation systems. This distribution of urban activities, consequently land use pattern and transport networks, largely determines the urban form and its related efficiency and livability. It is believed that accessibility can systematically bridge the transport system and the land use system components and, therefore, has been serving as a useful tool for urban and transport planning. The current study used data from the years 2012 and 2015. These data sets contained household travel survey data, transportation network, transit network, mobile phone signals, and land use data to develop the LUTI model.

The contribution of this study was to analyze the short-term TDA as dynamic/disaggregated for the locational preference of household and commercial urban activities, which could help to capture the impact of TDA under different time and transport network impedance conditions on the location choice of urban activities. However, the traditional accessibility terms are found to be inadequate to consider the short-term and long-term needs of urban activities. Therefore, it is important to find a solution that could provide a better planning tool to understand the urban system well. This study considered the dynamic short-term accessibility of urban activities and it could be a more useful tool in urban and transport planning compared to traditional “static” accessibility terms. On the other hand, the conventional location-based accessibility measures are static/aggregate and cannot reflect the accessibility fluctuation resulting from the differing performance of various modes (e.g., walking, cycling, car, bus, and metro) at different times of the day. In addition, this study used the PECAS model, which captures very fine details of how the decision of locating various urban activity generators (such as households, businesses, firms, etc.) are made. Although the methodologies that are applied to locational models are based on a diversity of attributes or characteristics, they share a particular common theoretical foundation based on the idea of maximizing utility. Furthermore, location models do not function in an isolated way; instead, they form a part of broader modeling systems’ LUTI modeling. It is essential to understand that the location choice of activities has a strong relationship with the transport system and is affected by time-dependent accessibility. Accessibility is a dynamic attribute of locations that varies over time and mode due to changes in the transport network and the varying distribution patterns of activities at different times in the day.

To validate the above hypothesis, the time-dependent TDM over 690 zones from the case study area was calculated using the ISE models, which were developed, calibrated, and validated across different cross-sectional years. Then, they were tested using the data from the case study area—the City of Wuhan. The TDM model was developed to measure the impact of short-term TDA on household and commercial activities during the morning peak, off-peak, and evening peak periods for the years 2012 and 2015. The ISE models were developed to evaluate the relationship between TDA and the choice behavior of activity locations within the study area for the years 2012 and 2015. The results from the ISE revealed that there was a strong relationship between TDA and the location choice behavior of urban activities.

After the above work is completed, the following important conclusions can be drawn from the results of this study. The results of the ISE models revealed that the location choice behavior of activities was very sensitive to a different type of TDA. For instance, the estimated activity location for the years 2012 and 2015 showed that when the accessibility increased during the morning peak and off-peak time, more activities were located in high accessibility locations. Household activities are susceptible to TDA during the morning peak, presumably related to their employment. Commercial activities are susceptible to TDA during off-peak, which indicates that shopping activities are preferably carried out during such periods at locations with good accessibility. The ISE models that are used in this study show a rich potential for evaluating different land use and transport policies with TDA. Without considering temporality, we may draw inferences based on inaccurate information. Dynamic accessibility with ISE modeling attempts to provide as realistic a general overview of spatial accessibility scenarios as is feasible, without extensive input on individual activity-travel behavior as in person-based models. This study serves as the foundation for developing advanced spatial and temporal accessibility modeling by presenting a general framework, demonstrating its applicability in practice, and presenting a set of potential spatial and temporal data sources. The study employed the PECAS model, which provided the precise location of activities by thoroughly evaluating a large data set. Therefore, policymakers will benefit from a more precise evaluation of spatial accessibility in urban areas.

### 6.2. Limitations and Recommendations

This study contributes additional accessibility-related insights to the literature. However, there are still a few shortcomings that must be considered in future studies. First, this study analyzed TDA with dynamic/disaggregate for urban activities. This study only considered the travel time, while ignoring the mode-based accessibility. Households with low income mostly rely on public transit to access their job locations as they cannot afford to own cars; conducting mode-based accessibility is advised to identify such an impact. It is also further advised to consider the hybrid accessibility, considering time- and mode-dependent accessibility to comprehensively capture the location preferences of household and commercial activities. In addition, to make the accessibility measures more inclusive, they must be modified to account for those factors which influence the location choice behaviors of various urban activities. This includes considering the following factors in studying accessibility: transport safety, security, comfort, reliability, and environmental sustainability.

With the above results and conclusions, several recommendations have been identified in this study. Future studies should consider the availability of opportunities at destination zones during different times of the day using advanced data sources such as social networking (Facebook, Twitter, etc.) or mobile phone signal data. Although this can be easily incorporated into the current models, the data availability makes it impossible for this study to carry out related analyses. High-fidelity land use models could be developed in the future that work at a very disaggregated level (such as land parcel) to produce more precise location choice models. In developing countries, mixed land use can be found everywhere. With a mixed land use plan, in most cases, the first few floors of buildings are allocated to commercial and office activities, and the remaining floors are allocated to residential activities. To obtain more robust and accurate results, a mixed land use model should also be added in future work.

## Figures and Tables

**Table 1:** Road network attributes.

Road Network	Description
Link type	Freeway, expressway, arterial, collector, and local
Distance	link distance (Km)
Road capacity	Daily and hourly capacity of each link
Lanes	Number of lanes in each link
AADTs	Average annual daily traffic counts collected at main road corridors
Screen lines	Screen line number associated with each AADT (used for model calibration)
Speed	Free-flow and congested speed

**Table 2:** Transit network attributes.

Name	Bus Name/Metro Name		
Time	Congested Time		
Distance	Distance in km		
Service frequency (minutes)	Modes	Metro	Bus
	Morning peak	3	3
	Off-peak	7	10
	Evening peak	5	5
Mode	Metro (Mode 1)		
	Bus (Mode 2)		
Line ID	Bus and metro line number		
Fare	Static fare for bus and distance-based for metro		
Waiting time	Initial waiting time and transfer waiting time at bus and metro station		
Other information		Metro	Bus
	Walking distance allowed (meters)	960	650
	Transfer distance allowed (meters)	550 (metro–bus, metro–metro)	550 (bus–metro, bus–bus)

**Table 3:** Time of the day considered.

Activities					
Production			Consumption		
Morning peak	Off-peak	Evening peak	Morning peak	Off-peak	Evening peak
√	√	√	√	√	√

**Table 4:** Locational preferences of activities parameters.

Activity Type	Commodity	Labor, Services, and Goods Type	Consume by	Transport Modes	Utility (Disutility)
Households	Labor	Management and technical labor	Industry and services	Auto, taxi, bus, metro	Logsum
		Retail service labor			
		Operators labor			
Commercial services	Services	Commercial services	Households and industry		

**Table 5:** Results of the TDA to household activities.

Year	2012			2015		
Time of Day	Morning Peak	Off-Peak	Evening Peak	Morning Peak	Off-Peak	Evening Peak
Residual Squares	5.997	7.816	7.161	5.341	6.402	6.560
R square	0.935	0.915	0.902	0.981	0.978	0.977
Adjusted R square	0.932	0.911	0.901	0.980	0.976	0.975

**Table 6:** Results of the TDA to commercial activities.

Year	2012			2015		
Time of Day	Morning Peak	Off-Peak	Evening Peak	Morning Peak	Off-Peak	Evening Peak
Residual Squares	69.62	68.32	70.63	9.501	8.693	9.371
R square	0.935	0.979	0.927	0.971	0.980	0.935
Adjusted R square	0.930	0.968	0.914	0.970	0.979	0.932

**Table 7:** TDA and urban activity location indexes.

	No	Low	Low-Medium	Medium	Medium-High	High
TDA (logsum)	0	0.22~0.92	0.93~1.13	1.14~1.28	1.29~1.39	1.40~1.51
Activity locations (density)	0	0.003~0.004	0.005~0.008	0.009~0.013	0.014~0.021	0.022~0.038

**Table 8:** Comparison between static and ISE-TDA models.

	Static Model		ISE-TDA Model	
Activities	Time of Day	R-Squared	Time of Day	R-Squared
**Households**	Daily	0.727	Morning peak	0.981
			Off-peak	0.978
			Evening peak	0.977
**Commercial**	Daily	0.485	Morning peak	0.971
			Off-peak	0.980
			Evening peak	0.935

## Data Availability

The data and concerning information may be obtained from the first author upon reasonable request.
